# It takes a village

**DOI:** 10.1172/JCI188177

**Published:** 2024-11-15

**Authors:** Alex D. Waldman

**Affiliations:** Emory University, Atlanta, Georgia, USA, National Institutes of Health, Bethesda, Maryland USA, and University of Oxford, Oxford, England, United Kingdom.

It is an honor to deliver the 2024 American Physician Scientists Association (APSA) Presidential Address in front of a group of such distinguished individuals. The talent in this room spans a variety of career stages and fields of biomedical pursuit. Importantly, we are all unified as we share one common goal: preserving the future of the physician-scientist, an entity that continues to be endangered (1). This partnership is what bonds us and represents our collective power. Collaboration is not merely a choice but a necessity. Collaboration begets community, which is essential to progress. Looking back at both my personal and professional life, the proverb “It takes a village” rings true not only for me but has also been essential for the growth and development of the APSA. I will use this time to parallel our journeys and highlight specific lessons learned.

The year was 1994. Power ballads topped the Billboard Charts, Beauty and the Beast opened on Broadway as the first stage adaptation of a Disney movie, and I was born to a Reform Jewish family in south Florida. Only upon reflecting back have I begun to understand how my “village” has shaped my identify and outlook. The traditional gender roles were reversed in my household. My mother was a human resources executive for Motorola who served as a staunch advocate for the use of blind resumes in hiring practices and implementation of unconscious bias training company wide. She could be considered the breadwinner of our family. Outside the corporate arena, she was also a sports fanatic and coached my brother’s middle school basketball team to victory. On the other hand, my dad was in the fashion industry. When not driving throughout the sunshine state showing off new styles of sunglasses to opticians, he had a penchant for watching trashy reality television. Beyond my nuclear family, Rocio and Arturo Blanco served as a second set of parents. They not only helped my parents raise me, but they were my windows into the Hispanic community of south Florida. They taught me the importance of learning about the diverse cultures present within the global melting pot, and this value has since been foundational. The unconventional structure of my familial unit, which went against the grain, unknowingly provided a foundational value system at home that not only accepted but also cherished diversity. In fact, this environment, and a protective and supportive older brother, enabled me to later embrace my LGBTQ+ identity.

Overall, I was shown that diversity goes beyond that of race and ethnicity but also includes gender, sexual orientation, religion, socioeconomic class, and culture. This broad view of diversity reflected in my childhood is a definition that APSA carries forward in its initiatives. In the wake of the Supreme Court decision regarding affirmative action, members of the Executive Council came together and wrote a commentary to outline mechanisms that should be implemented to diversify the physician-scientist workforce (2). These efforts include (a) increasing the number of dual-degree trainee positions, (b) expansion of early outreach programs, (c) strengthening mentorship structures, (d) improving access to formative research opportunities, and (e) implementing a transparent, holistic, and accessible application review process. APSA continues to brainstorm ways in which the community can increase the diversity found within the physician-scientist pipeline. After a successful inaugural Diversity Summit, we again collaborated with the Burroughs Wellcome Fund (BWF) to host an even larger event, and expect that our human-centered design approach will only further identify niches to intervene (3).

My village and the early lessons taught extend past the family that I saw daily during my childhood. My paternal grandmother, Lila Waldman, worked within institutions of higher education beginning in the 1940s. Beyond showing me the glitz and glamour of a Broadway show, she taught me the importance of relationship building and how events, such as the Joint Meeting, can serve as a forum for collaboration and fundraising. I distinctly remember her telling me in her typical Jewish Bubbie way, “If you don’t ask, you don’t get.”

APSA takes these ideals to heart. It is an honor to work closely with the Association of American Physicians (AAP) and American Society for Clinical Investigation (ASCI) to put on the Joint Meeting. Our collaborative relationship extends back to 2005, and the strength of our partnership only continues to grow with a bond that deepens year after year. I particularly want to share my deepest gratitude to the presidents of both societies for this past year, Dr. John Ioannidis and Dr. Benjamin Humphreys, as well as members of society management, Lori Ennis, John Hawley, Colleen McGarry, and Karen Guth, for their indelible support as I traversed this role and uncovered what it means to be a leader. APSA’s partnership footprint has also continued to grow. The BWF has awarded us a three-year longitudinal grant to support the Joint Meeting and our major organizational initiatives. Additional funding from the National Institutes of Health, American Association of Immunologists, American Society of Nephrology, American Society for Investigative Pathology, Society for Academic Emergency Medicine, and Foundation for Anesthesia Education and Research subsidizes trainee travel to the Joint Meeting. We are also particularly grateful for our continued collaboration with the Lasker Foundation. Beyond sponsoring the annual Lasker Laureate Lecture, this past year with Dr. David Huang, we collaborated on an additional event entitled “Interviewing an Icon.” This new session kicked off the meeting and served as an interactive opportunity to hear from a successful physician-scientist, Lasker Foundation Board Member Dr. Elizabeth Nabel, who has a storied career within academic medicine, government, health care administration, and industry. Our relationship only continues to blossom. Lasker has pledged to support the APSA Regional Meetings and will include APSA leaders as participants in the “Ask a Scientist” series to ensure the trainee voice is represented.

Turning back to my personal journey, a move to Chicago at 10 years old served as a pivotal moment of growth. It was the first time I had seen snow, but more importantly, it served as an opportunity to expand my village. We moved to Cary, Illinois. The demographics were in stark contrast to Coral Springs, Florida. In Coral Springs, I was immersed in a vibrant Jewish community. In Cary, I was the only Jewish person in school. However, I took this as an opportunity to share my culture with my classmates during Jewish holidays, and more significantly, through my Bar Mitzvah, which had the unique theme of “Alex’s Medical Center.” Beyond lasting friendships with my peers, I also forged strong relationships with my science teachers, Mrs. Therese Youel (AP Chemistry) and Mrs. Sandra Carlson (AP Biology), who nurtured my biomedical interests.

Going off to college at the University of Wisconsin–Madison marked another transformative experience. I was accepted into the Biology Core Curriculum (Biocore), an honors program led by Drs. Janet Batzli and Michelle Harris, with an alumni community that includes individuals like Lasker Awardee Dr. John Schiller. Biocore emphasized inquiry-based learning rather than rote memorization. “How do we know what we know?” was the motto. Laboratory sessions complemented didactic lectures and allowed students to drive the process of scientific discovery from experimental design to communication of findings via written and oral presentations. The program really emphasized the “guide on the side” mentality by providing reagents like transgenic C. elegans. This approach led to deeper understanding of foundational biological concepts. I subsequently joined Dr. Peter Ferrazzano’s laboratory, coupled with a course entitled Entering Research, led by Dr. Elaine Alarid. Dr. Ferrazzano and his colleague, Dr. Pelin Cengiz, started their labs as a joint effort to investigate different facets of neonatal stroke, as they both had the same mentor early in their training. Importantly, they shared key personnel and supported one another in securing grants. This collaborative model was my first experience in the scientific arena and was, to say the least, formative. In addition, Dr. Ferrazzano and Dr. Cengiz were both successfully pursuing the physician-scientist path as earlier stage investigators. They showed me there was a two-way crosstalk between lab work and clinical practice. When there is a missing link clinically, discovery-based research aims to elucidate pathological mechanisms and uncover possible interventions with hopes of effecting change. I ultimately learned that I wanted to be immersed on both sides of the crosstalk, not just the producing or receiving ends.

Medical school subsequently brought new challenges, new mentors, and a community of peers. From problem-based learning in small groups, led by clinician-educator Dr. Jason Schneider, to peer teaching anatomy labs with close friends, mentorship from those around me was essential to learning the essential foundations of medicine.

Overall, these personal educational experiences demonstrate the sheer importance of mentorship to identifying a career path and its subsequent pursuit. APSA realizes this and has been at the forefront of mentoring the next generation of physician-scientists via our undergraduate mentorship program, which enrolls over 300 diverse individuals each year. We continue pushing the envelope and have connected with the National Association of Advisors for the Health Professions to disseminate opportunities more widely to prospective trainees. We are also lucky to work with our strong collaborators, AAP and ASCI, to provide mentorship programming to current trainees. Dr. Dianna Milewicz, AAP Council member and APSA Board of Directors member, along with her colleagues, conceptualized the idea for a Physician Scientist Trainee Network. It has been an honor to work with her on a collaborative cross-society committee to launch robust mentorship programming at the Joint Meeting this past year. Fifty-four late-stage trainees (G4 and above) were ultimately assigned to 12 mentoring groups, each led by one AAP and one ASCI member in the trainee’s field of interest. We look forward to building up our community of mentorship families over the next six years and tracking how this intervention is able to mitigate the leaky physician-scientist pipeline with which we are all too familiar. In tandem, we assigned 122 earlier stage trainees into 22 groups for cross-sectional ad hoc mentorship at the Joint Meeting.

My graduate training represented a new journey that took me across the Atlantic. My research was conducted through the National Institutes of Health Oxford-Cambridge Scholars Program under the mentorship of the program’s founder, Michael Lenardo, and Gabriele De Luca, with invaluable support from the International Biomedical Research Alliance. This experience taught me that the fields of science and medicine are not discrete isolated units that fall in line with continental boundaries. Instead, these fields transcend national boundaries to create a dynamic and interconnected community of participants whose successes and failures hinge upon each other. For example, penicillin was discovered at the University of Oxford by Sir Alexander Fleming with the subsequent basic science elucidated by his colleagues. However, development and production of this new lifesaving antibiotic during World War II required collaboration with the United States Department of Agriculture and pharmaceutical companies (4). COVID-19 vaccine development and deployment represents an even more modern example of the intrinsic need for international collaboration to tackle difficult challenges (5).

APSA also understands the importance of the diverse global biomedical landscape. This deep understanding led to the development of the International Consortium of Clinician Scientist Trainee Organizations (ICCSTO) in 2019 (6). ICCSTO is a collaborative endeavor between APSA, Association Médecine Pharmacie-Sciences (AMPS), Clinician Investigator Trainee Association of Canada (CITAC), Asian Medical Students’ Association (AMSA), European MD/PhD Association (EMPA), and Swiss MD-PhD Association (SMSA). Since the inception of ICCSTO, we have compiled a comprehensive list of training programs for physician-scientists globally, including details regarding program structure and entrance requirements. This data-gathering initiative serves as the first step toward making essential comparisons in physician-scientist training worldwide while also generating a unified student voice for advocacy efforts.

I would be remiss if I didn’t take the time to thank the APSA Executive Council and members of the leadership team with whom I had the pleasure of working this past year. I was inspired daily by their dedication and tireless efforts. I also want to extend a warm welcome to the newly elected leadership, who I know will be just as passionate under the strong guiding hand of Dr. Cynthia Tang.

As you can see, there are many cinematic parallels between my journey and that of the APSA. I’d like to close with an analogy. Clinical work and discovery-based research fit together like pieces of a jigsaw puzzle. Piece by piece a jigsaw puzzle is constructed, each piece with its specific place equally as important as the other pieces to the overall scene it creates. Patient care is the big picture, the end result of the incredibly detailed piece-by-piece research conducted over the years. What does this mean for a career as a physician-scientist? Although clinically you can see an endpoint and accomplish it through assembling the already known pieces together, investigative research adds new pieces to the puzzle. However, a puzzle so complex begs for creative and collaborative solutions and your influential village is the key.

## Version 1 11/15/2024

Electronic publication

## References

[1] Wyngaarden JB (1979). The clinical investigator as an endangered species. N Engl J Med.

[2] Ding JL (2024). How to diversify the dwindling physician-scientist workforce after the US affirmative action ban. Nat Med.

[3] Rodrigues JA (2022). Climbing the ladder: toward a robust and diverse physician-scientist workforce. J Clin Invest.

[4] Gaynes RP (2017). Etymologia: Creutzfeldt-Jakob Disease. Emerg Infect Dis.

[5] Collins F (2023). The NIH-led research response to COVID-19. Science.

[6] Iness AN (2019). Waiting to “make it” versus “making it happen”: empowering physician-scientists in training. J Clin Invest.

